# An Unusual Case of Subclinical Peripheral Neuropathy and Cervical Spondylosis in Atopic Myelitis

**DOI:** 10.1155/2013/489451

**Published:** 2013-10-23

**Authors:** Alev Leventoglu, Pelin Ozlu, Ferda Ince

**Affiliations:** Department of Neurology, Ufuk University Medical School, Ankara, Turkey

## Abstract

Many cases of atopic myelitis have been reported in Japan; however very few were described in western countries. An 82-year-old woman with a past medical history of atopic dermatitis and asthma presented with progressive paresthesia (tingling) of both hands and tetraparesis. Before the onset of neurological symptoms, she complained of ichthyosis of both legs for 5 weeks. Magnetic resonance imaging demonstrated multisegmental degenerative arthritis, degenerative disc disease, and abnormal spinal cord signal intensity over several cervical segments, suggesting the diagnosis of myelitis. Total serum IgE level was elevated. Nerve conduction studies revealed asymmetric axonal sensorimotor neuropathy. The cerebrospinal fluid specimen showed lymphocytic pleocytosis and elevated protein level. Based on clinical, imaging, and laboratory findings, atopic myelitis was diagnosed. The diagnosis of atopic myelitis should be considered in myelopathy patients with history of atopy and elevated serum IgE levels.

## 1. Introduction

Atopy is characterised by an overactive immune response to environmental factors such as house dust mites, pollens, and foods and can be complicated with various neurological disorders, such as Hopkins' syndrome [1, 2], Hirayama disease [3, 4], idiopathic myelitis [5–8], peripheral neuropathy [9–11], and cervical spondylosis (intervertebral disc degeneration) [12]. These conditions are described to the association between allergic inflammation and central/peripheral nervous system disease and in part relate to the atopic backgrounds. In 1997 Kira et al. first reported four patients with cervical myelitis associated with atopic dermatitis (AD) and hyperIgEaemia, and they named the disease “atopic myelitis” (AM) [7]. The characteristic features of AM are (1) predominantly involvement of the cervical cord, as seen on MRI scans, (2) stepwise progression and fluctuation of the symptoms, (3) paresthesia/dysesthesia as the initial and predominant symptoms, (4) frequent coexistence with atopic dermatitis, (5) persistence of neurologic symptoms and MRI lesions (6) mild motor weakness, (7) normal or mildly cerebrospinal fluid (CSF) abnormalities, (8) absence of oligoclonal IgG bands and normal IgG index, (9) hyperIgEaemia, (10) presence of mite antigen-specific IgE, (11) mild peripheral blood eosinophilia, and (12) eosinophilic inflammation on spinal cord biopsy [6, 13, 14]

Most cases of AM have been reported in Japan since 1997, and nearly very few patients have been described in western countries [15–17]. We present a novel elderly case of atopic myelitis with mononeuropathy multiplex who had cervical spondylosis, hyperIgEaemia, and atopic dermatitis.

## 2. Case Report

A 82-year-old woman with a past medical history of atopic dermatitis and asthma for many years, but not remarkable disorder (atherosclerotic heart disease, hypertension, and trauma), sought medical treatment owing to 4-week history of progressive paresthesia (tingling) of both hands and tetraplegia. She complained of ichthyosis of the both legs for 5 weeks before the onset of neurological symptoms. Her family noted that she had a history of rhinoconjunctivitis and asthma after exposure to house dust mite, pollens, and irritating disinfectants. She had no familial history of neurological disorders. 

On presentation, she was alert with normal finding on examination of cognitive functions, cranial nerve, and speech. A general physical examination revealed skin lesions in her hands and frequently scratch the itchy skin in the proximal lower limbs. Neurologically, she had paresthesia on both palms and hyperreflexia in her lower limbs with flexor plantar responses. The bilateral forearm and hand muscles demonstrated moderate weakness without atrophy, and there was stiffness of the fingers and difficulty in relaxing her hand grip. Lower extremity motor examination revealed moderate weakness, increased tone, and strength. Sensory examination was normal. No sphincter disturbance was found. 

Routine laboratory tests including haemogram, creatin kinase, urinalysis, serum vitamin B12 level, thyroid function test and folate, and angiotensin-converting enzyme levels were normal. HIV testing (ELİSA) was negative. The oral glucose tolerance test showed a normal pattern. The C-reactive protein level was 11.41 mg/L (0.01–5.00), and sedimentation rate was 26 mm/hr, indicating inflammation. Her cerebrospinal fluid (CSF) showed mild pleocytosis (10/mm^3^, mononuclear cells), slightly raised protein (95 mg/dL), and mild decreased glucose. CSF IgG index was normal and there was absence of oligoclonal bands. However, Lyme, syphilis, and *Brucella* serologic tests results were negative in cerebrospinal fluid and serum. No abnormality was noted in the coagulation/bleeding system including factor 13. The tuberculin test was not performed.

**Figure 1:**
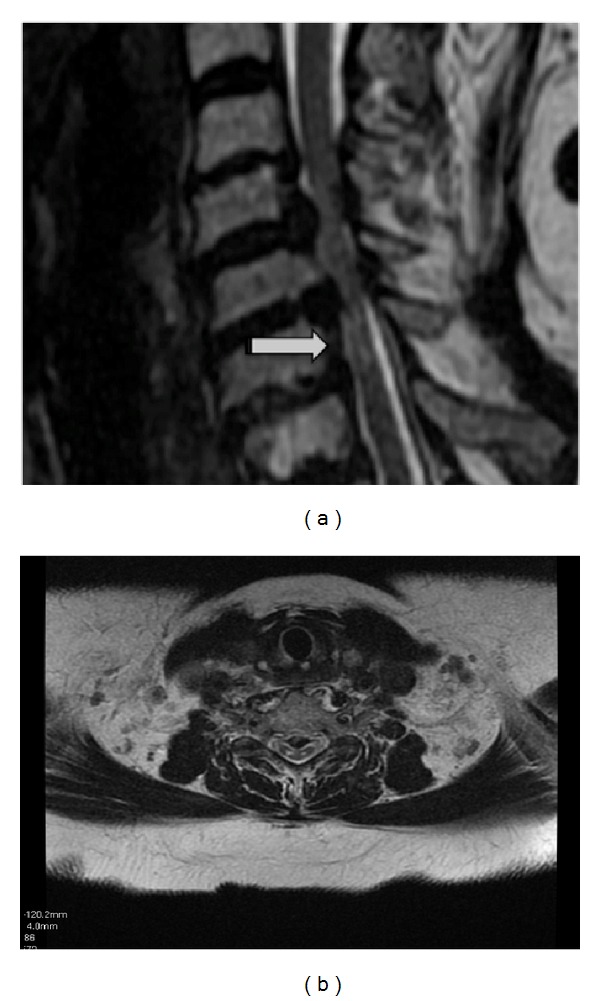
(a) and (b): T2-weighed MRI on sagittal and axial plans of spinal cord linear nonenhancing high signal intensity lesions.

Magnetic resonance (MR) imaging of the brain, including T1, T2, fluid attenuated inversion recovery, and gadolinium, enhanced T1-weighed images, and was normal. Cervical spinal MRI demonstrated multisegmental degenerative arthritis and degenerative disc disease, especially C5–C7 stenosis, and multisegmental bilateral foraminal encroachment at C4-C5 and C5-C6, as well as C6-C7 narrowing. T2-weighed images revealed linear, nonenhancing high signal intensity lesions, mainly affecting the posterior column consistent with chronic spondylotic myelopathy (Figures 1(a) and 1(b)). Lumbar and thoracic cord spinal MRI were normal.

The first electrodiagnostic studies including right median, ulnar, bilateral common peroneal, posterior tibial, and sural nerve were normal. Electromyography of the right biceps, iliopsoas, and abductor pollicis brevis muscles demonstrated spontaneous activity as well as fibrillation, positive sharp wave, and complex repetitive discharge. Chronic neurogenic motor unit potentials were evident in the right vastus lateralis muscle. Repetitive nerve stimulation was applied to right abductor digiti minimi and orbicularis oculi muscles. Electromyography revealed an incremental response of greater than 49% in the abductor digiti quinti muscle on 20 Hz. However, no decremental response was observed on 2, 3, and 5 Hz stimulation in the orbicularis oculi muscle and abductor digiti quinti muscles (Table 1). Acetylcholine receptor (AchR) binding antibody titers, chest X-ray, abdominal ultrasound, bilateral mammography, and computerized torax tomography were normal. Serum levels of the tumor markers such as carcinoembryonic antigen (CEA), carbohydrate antigen 19-9 (CA 19-9), alpha fetoprotein (AFP), Ca 15-3, Ca-125, and Ca 72-4 became normal. Voltage gait calcium canal antibodies were negative. Serum IgG, IgA, IgM, C3, and C4 were normal as well as rheumatoid factor, anti-double stranded DNA antibody, anti-SSA and SSB antibody, antinuclear antibody, and anti-Sm/RNP. Blood tests revealed an elevated serum IgE of 3757 IU/mL (normal range 0.01–180.00) and an eosinophil count of 9.5% (normally 0.9–2.9%). Skin prick testing showed strong positive reactions to multiple antigens, including house dust mite, pollen, and hydrochloric acid.

The patient was started on plasmapheresis soon after admission. Although she had an allergic reaction including oral edema dysphagia during plasmapheresis, she received plasmapheresis for three time, and pulse therapy with methylprednisolone of 1,000 mg/day for 3 days, followed by tapered oral steroids and antihistaminic for 2 months. Afterwards intravenous administration of methylprednisolone and plasmapheresis slightly improved the patient's paresthesia and general motor function. 

Repeat electrophysiological studies were applied to right and left median, ulnar, sural, common peroneal, and tibial nerve. The electrodiagnostic studies showed the following abnormalities (Table 1): reduction of the amplitude of the M responses in the bilateral peroneal nerves, right tibial and median nerves; reduction of sensory nerve action potential and conduction velocity in the digit V; reduction of sensory nerve action potential amplitude in the right digit-palm, bilateral palm-wrist, wrist-elbow in median nerve, and wrist-elbow segment in right ulnar nerve; and bilateral prolonged peroneal *F*-waves. There were normal findings in the left median, bilateral ulnar, left tibial, and bilateral sural nerves. Needle EMG revealed positive sharp waves, fibrillation potentials in the right tibialis anterior, iliopsoas, abductor pollicis brevis, abductor digiti quinti, biceps, and reduced recruitment in the right iliopsoas muscle. After plasma exchange, a repeat EMG did not show complex repetitive discharge or pseudomyotonia from the initial electrophysiological findings.

Biopsy of the sural nerve and the peroneus brevis muscle was performed. The nerve specimens were prepared for light and electron microscopy. The frozen muscle sections were stained with hematoxylin-eosin, modified Gomori's trichrome, and a battery of histochemical stains. No cellular infiltration was found in the endoneurium or around the epineural blood vessels.

## 3. Discussion

We presented here an 82 year-old woman patient with acute myelitis due to a cervical spinal lesion with background intervertebral disc degeneration. Her clinical or laboratory findings did not match the intervertebral disc degeneration. Our electrophysiological data showed diffuse acute denervation, pseudomyotonia, and complex repetitive discharges. On the basis of these findings, we suspected the presence of paraneoplastic syndromes such as LEMS or channelopathies in our patient. The repetitive nerve stimulation test showed probable presynaptic neuromuscular transmission deficit. However, brain MRI, K channel antibody, Ach receptor antibody, and CSF were normal. The patient did not have a history of cervical injury or trauma, but she suffered from atopic dermatitis and asthma with house dust mites, pollens, foods, and detergents for many years. 

Atopic myelitis is a rare occurrence with only 3 cases reported in western countries [15–17]. AM considers myelitis in association with atopic disorders, such as bronchial asthma, eczema, or atopic dermatitis. It is defined as raised serum total IgE and raised IgE titers to common environmental antigens. The myelitis usually affects young to middle age adults. Characteristic clinical features include acute onset, paresthesia/dysesthesia in the distal parts of four limbs, and mild motor disability. The disease predominantly affects the posterior column of the cervical cord, of which approximately 50% show contrast enhancement on MRI [13]. However, the exact etiology of AM has been unclear. 

Atopic disorders usually develop in patients with an individual or family history of allergic diseases and are characterized by chronic relapsing inflammation seen in patients, in association with IgE hyperproduction and precipitation by environmental antigens. Many investigators accept that the binding of allergens to mast cell-bound IgE initiates the release of vasoactive amines and inflammatory mediators [18]. IgE-mediated mast cell activation leads to release of various kinds of chemical mediators (e.g., histamine), lipid metabolites (e.g., prostaglandins), cytokines, and chemokines which results in infiltration of inflammatory cells into the skin lesion, such as eosinophils and lymphocytes [19]. Activated Th2 lymphocytes produce IL-4, IL-13, and IL-5, which are responsible for IgE production by B cells, eosinophil activation and recruitment, and mucus production. On the other hand, Th1 T cells known as predominantly produce IFN*γ* and delayed type hypersensitivity, while Th2 secrete IL-4, IL-5, IL-6, and IL-13 and regulate B cell and eosinophil mediated responses [20]. In patients with AD decreased production of IFN-*γ* is considered to be associated with IgE hypersynthesis and Th2 immune responses [21]. Therefore, myelitis of unknown etiology, particularly myelitis with atopic disorders, is considered to have a Th2 shift in peripheral blood of onset of neurological symptoms. Thus, the binding of allergens to mast cell bound IgE initiates the release of inflammatory mediators and mast cell may well cause a breakdown of the blood-brain barrier (BBB). Deposition of activated eosinophil products as well as infiltration of eosinophils in the spinal cord lesions suggests the local production of eosinophil chemoattractants, such as IL-5 [14].

Spondylosis progresses with age and often develops at multiple interspaces. The preferential involvement of the cervical cord in myelitis with AD has yet to be explained. Ito et al. [12] reported the potential association of cervical spondylosis (intervertebral disc degeneration) with AD. Their 65 patients with AD showed intervertebral disc degeneration in 32 (86%), disc bulging/protrusion in 29 (78%), posterior spondylolisthesis in 24 (65%), kyphosis in 11 (30%), and thickening of yellow ligaments in two (5%). Degenerative disc disease is a spinal condition caused by the breakdown of intervertebral discs. Intervertebral disc degeneration is considered to be mainly caused by mechanical stress, aging, injury, arthritis, and osteoporosis. Spondylosis progresses with age and often develops at multiple interspaces. Chronic cervical degeneration is the most common cause of progressive spinal cord and nerve root compression. Horiuchi et al. [22] and Ito et al. [12] showed that Th2 dominant inflammation contributes to disc degeneration in AD patients. AD may be one of the risk factors for intervertebral disc degeneration.

Exposure to substances in the workplace causes more than 10% of all cases of adult-onset asthma [23]. Most cleaning agents have an irritating effect on mucous membrane and the skin, and occasionally a sensitizing potential. These agents associated with asthma-like symptoms have harmful irritative and/or sensitizing properties and may be involved in the development of chronic respiratory symptoms [24]. The surfactants from detergents, such as fatty acid salts (soap) and organic sulphonates, and hydrochloric acid could interfere with IgE-mediated sensitization to ubiquitous enviromental allergens. Poulsen et al. have hypothesized that the strong surfactant properties of some ingredients of modern detergents may interfere with various intricate cellular interactions taking place along immunological pathways, including formation of Th2 T cytokine [25].

It has been suggested that electrophysiological findings, such as complex repetitive discharges, pseudomyotonia, fasciculation, and fibrillation, occur as a result of deposition of activated eosinophil products and infiltration of eosinophils in the spinal cord lesions strongly suggests the local production of the eosinophil chemoattractants. In addition, it is possible that eosinophils infiltrating from the peripheral blood into the spinal cord may contribute to the development of inflammation [6]. Osoegawa et al. [10] reported that subclinical PNS involvement associated with AM. They reported that there were three patterns of neuropathy in AM patients: axonal neuropathy, compression neuropathy, and demyelinating neuropathy, [10]. In our patients, we showed asymmetric axonal sensorimotor neuropathy and axonal neuropathy affecting multiple peripheral nerves in the AM patients. Inoue et al. reported the patients with ChurgeStrauss syndrome (allergic granulomatous angiitis-AGA) and mononeuritis multiplex showed an increase in deposition of IgE and proposed that allergic mechanism may contribute to the development of peripheral nerve injury in AGA [26].

Finally, our patient is the first reported case of atopic myelitis with mononeuropathy multiplex who had cervical spondylosis, hyperIgEaemia, and atopic dermatitis in outside Japan. In our country the prevalence of atopic myelitis is rarely compared to those of Asian countries. Consequently, failure to diagnose AM or its misdiagnosis leads to advanced disease making treatment difficult and prognosis poor. Taking a good history, examining patients, and measuring total and specific IgE levels should be done for diagnosis of atopic myelitis. Further studies are needed to determine the incidence of the atopic myelitis, its prevalence in western countries, and to understand mechanisms of pathogenesis and the clinical significance.

## Figures and Tables

**Table 1:** Series studies in motor and sensory nerve conduction studies.

Motor nerve conduction studies (NCS)																	Sensory NCS		
	Median (M)				Ulnar (U)				Peroneal				Posterior tibial				M	U	Sural
	dL (ms)	Amp (mV)	CV (m/s)	F (m/s)	dL (ms)	Amp (mV)	CV (m/s)	F (m/s)	dL (ms)	Amp (mV)	CV (m/s)	F (m/s)	dL (ms)	Amp (mV)	CV (m/s)	F (m/s)	Amp (*μ*V)	Amp (*μ*V)	Amp (*μ*V)
Reference limit																			
	<3.8	>4.3	>49	>32	<3.3	>7.0	>49	>32	<5.8	>3.6	>40	>52	<5.8	>3.6	>39	>52	>10	>7.0	>5.0

The first electrodiagnostic studies (before plasmapheresis)																			
R	3.4	8.6	50.0	25.5	2.3	11.0	60.0	23.0	3.4	3.8	45.0	43.0	5.2	5.5	43.0	45.0	12.0	8.8	8.5
L	3.3	7.0	48.0	25.0	2.2	10.0	59.0	22.0	3.7	3.9	50.0	40.0	4.2	5.9	42.0	47.0	11.0	8.5	8.0

The second electrodiagnostic studies																			
R	3.6	**2.8**	51.1	29.0	2.1	7.2	57.0	28.0	3.8	3.8	45.0	**58.8**	4.9	3.8	43.0	49.0	**4.1**	**3.4**	9.0
L	3.1	5.7	53.8	28.0	2.1	**6.4**	66.7	27.0	3.6	**2.6**	45.0	**54.0**	4.2	5.9	42.0	52.0	10.0	8.0	10.0

R: right, L: left.
